# A Niche-Based Framework to Assess Current Monitoring of European Forest Birds and Guide Indicator Species' Selection

**DOI:** 10.1371/journal.pone.0097217

**Published:** 2014-05-12

**Authors:** Amy S. I. Wade, Boris Barov, Ian J. Burfield, Richard D. Gregory, Ken Norris, Petr Vorisek, Taoyang Wu, Simon J. Butler

**Affiliations:** 1 Centre for Agri-Environmental Research, School of Agriculture, Policy and Development, University of Reading, Reading, United Kingdom; 2 BirdLife Europe, Brussels, Belgium; 3 BirdLife International, Cambridge, United Kingdom; 4 The Royal Society for the Protection of Birds, The Lodge, Sandy, Bedfordshire, United Kingdom; 5 Czech Society for Ornithology, Prague, Czech Republic; 6 Department of Zoology and Laboratory of Ornithology, Faculty of Science, Palacký University, Olomouc, Czech Republic; 7 School of Computing Sciences, University of East Anglia, Norwich Research Park, Norwich, United Kingdom; 8 School of Biological Sciences, University of East Anglia, Norwich Research Park, Norwich, United Kingdom; University of Fribourg, Switzerland

## Abstract

Concern that European forest biodiversity is depleted and declining has provoked widespread efforts to improve management practices. To gauge the success of these actions, appropriate monitoring of forest ecosystems is paramount. Multi-species indicators are frequently used to assess the state of biodiversity and its response to implemented management, but generally applicable and objective methodologies for species' selection are lacking. Here we use a niche-based approach, underpinned by coarse quantification of species' resource use, to objectively select species for inclusion in a pan-European forest bird indicator. We identify both the minimum number of species required to deliver full resource coverage and the most sensitive species' combination, and explore the trade-off between two key characteristics, sensitivity and redundancy, associated with indicators comprising different numbers of species. We compare our indicator to an existing forest bird indicator selected on the basis of expert opinion and show it is more representative of the wider community. We also present alternative indicators for regional and forest type specific monitoring and show that species' choice can have a significant impact on the indicator and consequent projections about the state of the biodiversity it represents. Furthermore, by comparing indicator sets drawn from currently monitored species and the full forest bird community, we identify gaps in the coverage of the current monitoring scheme. We believe that adopting this niche-based framework for species' selection supports the objective development of multi-species indicators and that it has good potential to be extended to a range of habitats and taxa.

## Introduction

The majority of European forests are semi-natural ecosystems, heavily influenced by anthropogenic management and exploitation [1],[2]. Managed and disturbed forests tend to have lower biodiversity than primary forest [3] and there is mounting concern about the state of Europe's forest wildlife, with the populations of many forest species in decline [1], [4]. Accordingly, efforts to mitigate the negative impacts of intensive forest management, through changes in European and national policies, improved targeting of biodiversity management actions and certification of sustainably managed forests, have increased [5], [6]. To assess the success of resultant modifications to management practice in counteracting the detrimental impacts of existing or emergent drivers of biodiversity decline, appropriate monitoring of forest ecosystems is paramount.

Ecological indicators are a useful and widely adopted tool for assessing biodiversity health and ecological change [7], [8]. They measure trends of a proportion of the ecological community with the aim of providing a representative portrayal of the state of the wider community. The characteristics of a good indicator have been widely discussed and a number of key attributes have been identified [8], [9]. A good indicator should i) be representative, reflecting the status of the wider community; ii) be reactive, acting as an early warning system to ecological change; iii) respond to change in a predictable way; and iv) be straightforward to compile, analyse and interpret. The most crucial aspect of indicator development that ensures these key attributes are met is selecting the species to be included. Multiple methods of species' selection have been adopted but these often rely on expert opinion [10] or statistical methods that require population data and can be site specific [11]. Generally applicable and objective methodologies for species' selection are frequently lacking, as are methods for assessing whether existing indicators are fit for purpose.

Butler *et al*. [12] presented an objective, niche-based approach to the selection of indicator species that facilitates the formation of an indicator with all of the aforementioned key attributes. However, the structure of the search algorithm used to support the approach meant that, due to associated computational demands, the number of candidate species from which the indicator set could be drawn was restricted to approximately twenty-five. Here we present a new search algorithm (SpecSel), based on the same conceptual framework but that overcomes these limitations and facilitates application of this approach to much larger communities. We use it to select species for inclusion in a pan-European forest bird indicator. Birds have been the primary focus for many terrestrial applications of multi-species indicators in the UK and Europe [7], [13], with indices of wild bird population trends being adopted as indicators of sustainable development and as proxies for wider environmental health and human well-being [14], [15]. Specifically, we i) select a set of species that together possess all the key attributes of a good indicator and whose selection follows a repeatable and generally applicable objective method, ii) contrast this with an existing pan-European forest bird indicator (http://www.ebcc.info/index.php?ID=459) which comprises 33 species selected on the basis of being abundant, widespread and characteristic of forests according to expert opinion [7] and iii) explore a number of alternative indicators, suited to more specific monitoring objectives. In doing so, we assess the coverage of current bird monitoring schemes and identify key gaps.

## Materials and Methods

Butler *et al*. [12] provide a detailed methodology of the niche-based species' selection protocol. In brief, a list of candidate indicator species is drawn up and their niche space coarsely defined in the form of a matrix of resource requirements. Depending on existing knowledge about the habitat for which the indicator is being developed, this list could be derived from, for example, a literature search, species' distribution maps and/or baseline surveys. Each species' reliance on the specified habitat to provide its key resources is also categorized. A set of indicator species is then selected from this initial list on the basis of two principal rules: 1) all resource types used by the wider community must be exploited by at least one species included in the indicator species' set; and 2) the indicator species' set must comprise the most specialised species possible; more specialised species are taken to be more sensitive to changes in resource availability [16], [17]. Adherence to these rules ensures that the indicator is representative because the niche space occupied by the selected species fully encompasses that occupied by the wider community whilst maximising the indicator's sensitivity to changes in resource availability. Each species' sensitivity to changes in resource availability is calculated as its niche breadth multiplied by its reliance, with niche breadth defined as the number of resource types it exploits and reliance scored as major  =  1, moderate  =  2 and minor  =  3. Higher scores indicate less sensitive species, which are assumed to be less susceptible to changes in the availability of resources [18].

### Candidate species selection and resource quantification

A pool of 80 candidate forest indicator species was identified, including all species categorised as having >10% of their breeding population using European forest habitat [19], with a relatively extensive range (present in ≥5 countries [20]) and which were included in at least one of nine key community-wide, pan-European or pan-regional studies [4], [11], [21], [22], [23], [24], [25], [26], [27]. The resource requirements matrix was constructed by broadly categorising each species' summer and winter diet, summer and winter foraging habitat and nest site location (see Tables S1, S2, S3), reflecting readily available information on their ecology and natural history [20]. In addition, species' reliance (major, moderate or minor) on forest habitat to provide these resources was independently scored by 49 ornithological experts from 20 European countries, with no prior knowledge of the study. Modal responses were calculated by country and the modal response across countries was used in analyses (Table S4).

### Identifying candidate species' sets

The new species' selection algorithm (SpecSel) is based on the concept of minimal dominating sets [28]. Here an indicator set is called minimal dominating if the particular species' combination satisfies Rule 1 (complete resource coverage) and if the removal of any species from it causes a loss of complete resource coverage; every species' combination satisfying Rule 1 is either a minimal dominating set or contains at least one such set as its subset. In brief, the algorithm employs a search tree data structure [29], using a data reduction rule based on a matrix of resource use associations within and between candidate species, to enumerate all minimal dominating sets for each indicator set size.

**Figure 1:**
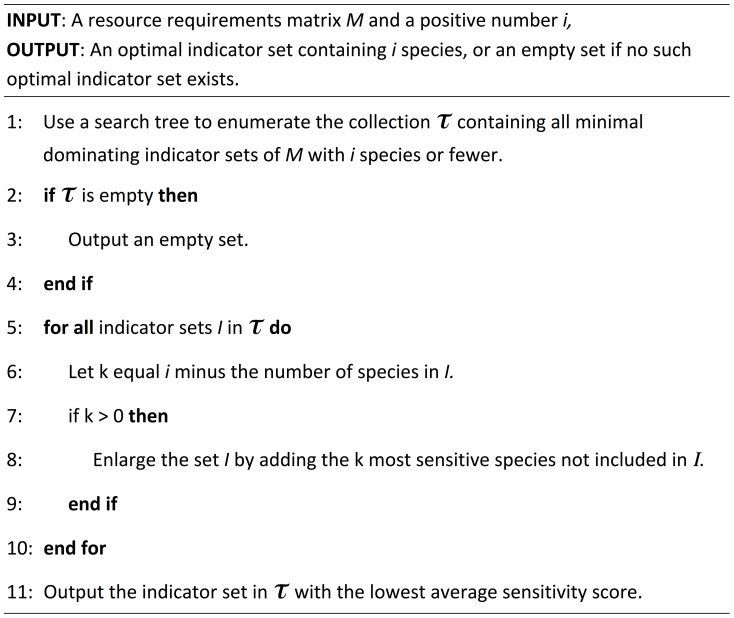
Overview structure of SpecSel, the species' selection algorithm, outlining the process to identify the optimal indicator set for each set size. SpecSel has been implemented in Java and the program, including detailed coding for the search tree component, can be freely downloaded from https://www.uea.ac.uk/computing/specsel.

To identify the optimal species' combination for a given indicator set size *i*, SpecSel compares the average sensitivity score of all minimal dominating sets containing *i* species with that of the minimal dominating set(s) containing *i*-1 species plus the single most sensitive species not included in that set, the minimal dominating set(s) containing *i*-2 species plus the two most sensitive species not included etc (Fig. 1). Data processing time depends on the number of species included in the candidate pool, the number of resources used by candidate species and the degree of overlap in resource use between species, with larger candidate pools or those containing more specialised and/or more diverse species taking longer to resolve. As a guide, resolution of the pan-European indicator species' selection, drawing from the pool of 80 candidate species, took approximately five minutes but the broadleaf-dominated forest indicator, which drew from a candidate pool of only 69 species, took over an hour to resolve because of greater niche-partitioning (i.e. lower resource use overlap) across species. SpecSel has been implemented in Java and the program can be freely downloaded from https://www.uea.ac.uk/computing/specsel.

### Identifying optimum combination across set sizes

For each indicator set size, the algorithm described above identified the species' combination(s) that best met the two selection rules; for some set size categories, more than one combination had the lowest average sensitivity score because some species had the same sensitivity scores and were interchangeable. A previous application of this approach to UK farmland birds [12] suggested that average sensitivity scores decline (i.e. the indicator becomes more sensitive) as indicator set size increases, at least for smaller set sizes, but that the rate of change decreases. For each indicator type we therefore present three alternative species' sets – i) the set with the fewest species (hereafter *MINIMAL*), ii) the set with the lowest average sensitivity score (hereafter *SENSITIVE*) and iii) the set identified by piecewise regression as the optimal breakpoint when relating indicator set size to average sensitivity (hereafter *BREAKPOINT*) – and discuss the relative merits of each.

### Additional indicators

We also identified indicator combinations for several alternative contexts, applying the same procedure described above to each but excluding species and/or resources not relevant to the specific habitat type or community from the initial requirements matrix and species' pool. Firstly, we derived regional indicator sets for four regions of Europe (North, East, South and West as defined by the Pan-European Common Bird Monitoring Scheme (PECBMS)). The PECBMS is an association of experts and national organizations cooperating through the European Bird Census Council (EBCC) and BirdLife International, with technical assistance from Statistics Netherlands. This scheme collates population trend data from annually operated national breeding bird surveys from across Europe to generate supra-national trends; indices based on these data have been taken up as official statistics of biodiversity health and sustainable development by the European Union [7], [13]. For regional indicators, only species with >5% of their European breeding population in that region were included and only reliance scores from countries within that region were used to calculate species' sensitivity (Table S4). We also derived indicator sets specific to conifer- and broadleaf-dominated forests, with only species recorded as using the target forest type included in its initial species' pool. To calculate species' sensitivity scores for these indicators, niche breadth was quantified as the number of resource types used by a specific species in the target forest type. Forest-type specific reliance scores were not available so were calculated from species' overall reliance scores and the number of forest types they used; a species' reliance score was doubled if it uses both conifer- and broadleaf-dominated forest but kept the same if found in only one forest type. This adjustment is based on the assumption, in line with the wider conceptual framework, that a species found in both forest types would be less sensitive to changes in resource availability in the target forest type, and therefore a less appropriate indicator species for it, than one found only in the target forest type (). Note therefore that sensitivity scores for the forest-type indicators are not directly comparable to those for other indicators presented here. For the pan-European, regional and forest-type indicators, we also derived comparable indicators drawn only from species that are currently covered by PECBMS. For all additional indicator types we again present the three alternative species' combinations as discussed above.

### Assessing indicator characteristics

Using equivalent methods to these used to calculate existing pan-European and regional indices [13], [30], we used national population data between 1980 (1982 for East and 1989 for South) and 2011 to calculate annual index values for all indicator sets described above. For each indicator type, i.e. pan-European, regional and forest-type, we used inter-annual changes to compare the temporal dynamics of alternative indices (*MINIMAL*, *BREAKPOINT*, *SENSITIVE* and, for the pan-European and regional indicators, existing multi-species indicators) to that of an index based on the population trends of all species in the candidate pool from which the sets had been drawn (hereafter *COMMUNITY*). We expected a significant positive relationship between the inter-annual changes of each indicator and *COMMUNITY* and that the scale of change would be greater for our indicator sets than for the *COMMUNITY* i.e. the slope of any regression with inter-annual change in *COMMUNITY* as the dependent variable and the inter-annual change of one of our indicators as the predictor variable would be less than one. To test these, Type II major axis regression, implemented in the ‘*lmodel2*’ package in R [31], was used as there may be error in both the *x* and *y* variables [32]. Significance of the correlation coefficient was assessed using 9999 permutations. Note, these comparisons were only possible for indicators drawn from species currently covered by PECBMS.

## Results

Between them, the 80 species that met our criteria for inclusion in the candidate species' pool covered 191 resource types (Tables S1, S2, S3). Of these, 58 species are currently covered by PECBMS, together exploiting 172 of the 191 resources types used by the full community. Thirty of the 33 species included in the current pan-European forest bird indicator were included in our initial pool; *Bombycilla garrulus*, *Cyanopica cyanus* and *Tringa ochropus* did not meet our inclusion criteria. Between them, these 30 species covered 164 of the 191 resource types used by the full community.

### Pan-European forest bird indicator

**Figure 2:**
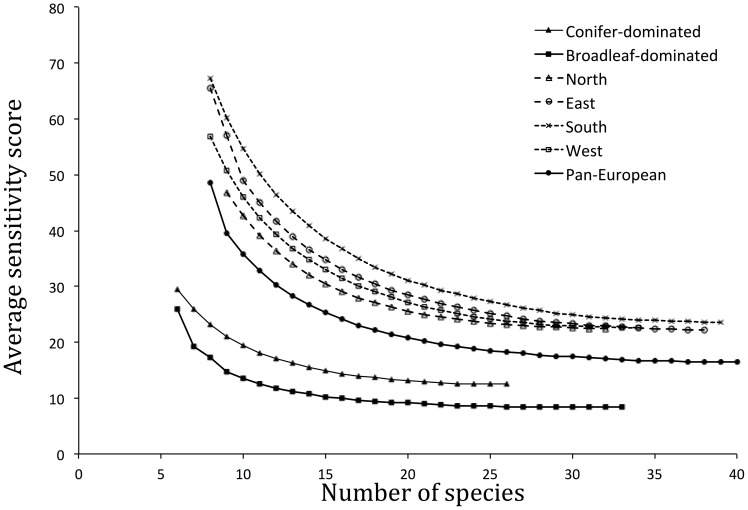
Relationship between the number of species in the indicator and the average sensitivity score of constituent species in the most sensitive combination for that set size for the pan-European and alternative indicators drawn from all possible species. Average sensitivity scores calculated as average of niche breadth*reliance across constituent species, with higher scores associated with less sensitive indicators. See Figure S1 for the equivalent figure for pan-European and alternative indicators drawn only from species currently covered by PECBMS.

*MINIMAL* contained eight species, with an average sensitivity score across constituent species of 48.5 (Table 1). The average sensitivity score of the optimal combination within each indicator set size category decreased with increasing indicator set size to a minimum value of 16.4 for *SENSITIVE*, which included 40 species (Fig. 2, Table S5); beyond this, average sensitivity scores increased and the indicator became less sensitive with increasing set size. Piecewise regression identified a breakpoint in the relationship between indicator set size and average sensitivity score at 15 species, with a break here providing a significant improvement to the basic linear model (F_2, 29_  =  130.7, P < 0.001; Table 2). All species included in *MINIMAL*, except the least sensitive species *Columba palumbus*, were also included in this *BREAKPOINT* set. Of the 33 species included in the current pan-European forest bird indicator, only two were included in *MINIMAL*, 6 in *BREAKPOINT* and 16 in *SENSITIVE* (Tables 1, 2 and S5).

**Table 1:** Species included in the *MINIMAL* sets for the main pan-European indicator (Main), the equivalent indicator drawn only from species currently covered by PECBMS (Main-PECBMS) and the forest-type specific and regional indicators.

Species	Main	Main-PECBMS	Conifer-dominated	Broadleaf-dominated	North	South	East	West
Community size	80	58	54	69	40	61	68	58
Total resources	191	172	66	62	191	190	190	190
*Accipiter gentilis*	1			1	1	1	1	1
*Coccothraustes coccothraustes* *	1	1		1		1	1	1
*Columba palumbus*	1	1		1	1	1	1	1
*Dendrocopos major*	1	1	1		1	1	1	1
*Nucifraga caryocatactes* *	1	1	1		1	1	1	1
*Turdus merula*	1	1	1		1	1	1	1
*Troglodytes troglodytes*	1	1	1	1		1	1	1
*Tetrao urogallus*	1		1		1			
*Accipiter nisus* *		1						
*Buteo buteo*		1						
*Parus cristatus* *		1				1	1	1
Aegolius funereus			1					
*Dendrocopos leucotos*				1				
*Picus canus* *				1				
*Cuculus canorus*					1			
*Garrulus glandarius* *					1			
*Parus montanus* *					1			
Number of species	8	9	6	6	9	8	8	8
Maximum sensitivity score	108	108	72	52	108	162	120	120
Average sensitivity score	48.50	45.89	29.50	26.00	46.89	67.25	65.50	56.75

Species' sensitivity scores are calculated as their niche breadth*reliance, with higher values indicating species less sensitive to changes in resource abundance or availability. Equivalent, PECBMS-only *MINIMAL* sets for the forest type and region indicators are presented in Table S6.

*Species also included in current pan-European forest bird indicator (for full list see http://www.ebcc.info/index.php?ID=459).

**Table 2:** Species included in the *BREAKPOINT* sets for the main pan-European indicator (Main), the equivalent indicator drawn only from species currently covered by PECBMS (Main-PECBMS) and the forest-type specific and regional indicators.

Species	Main	Main-PECBMS	Conifer-dominated	Broadleaf-dominated	North	South	East	West
*Accipiter gentilis*	1			1	1	1	1	1
*Coccothraustes coccothraustes* *	1	1		1		1	1	1
*Dendrocopos major*	1	1	1		1	1	1	1
*Dendrocopos medius* *	1	1		1		1	1	1
*Emberiza rustica* *	1			1	1			
*Ficedula albicollis* *	1	1						
*Loxia curvirostra*	1		1		1	1	1	1
*Loxia pytyopsittacus*	1		1		1			
*Nucifraga caryocatactes* *	1	1	1		1	1	1	1
*Oriolus oriolus*	1	1		1		1	1	1
*Pyrrhula pyrrhula* *	1	1		1		1	1	
*Tetrao tetrix*	1			0/1^a^				
*Tetrao urogallus*	1		1		1			
*Troglodytes troglodytes*	1	1	1			1	1	1
*Turdus merula*	1	1	1		1	1	1	1
*Phoenicurus phoenicurus* *				0/1^a^				
*Accipiter nisus* *		1						
*Bonasa bonasia* *		1					1	
*Buteo buteo*		1						
*Columba oenas* *		1				1	1	
*Parus cristatus* *		1				1	1	1
*Phylloscopus bonelli* *		0/1^a^	1			1		
*Phylloscopus sibilatrix* *		0/1^a^	1	1	1	1	1	1
*Aegolius funereus*			1					
*Picoides tridactylus*			1		1			1
*Ciconia nigra*			0/1^b^					
*Aquila pomarina*			0/1^b^					
*Regulus ignicapilla* *			0/1^b^					
*Garrulus glandarius* *				1	1			
*Parus palustris* *				1				
*Picus canus* *				1				
*Columba palumbus*					1			1
*Cuculus canorus*					1			
*Muscicapa striata*					1	0/1^c^	0/1^a^	1
*Picoides tridactylus*							0/1^a^	
*Parus montanus* *					1			
*Sylvia borin*					1			
*Ficedula hypoleuca* *						0/1^c^		1
*Luscinia megarhynchos*						0/1^c^		
*Phylloscopus collybita* *						1		
*Jynx torquilla*								1
Number of species	15	15	12	11	16	17	15	16
Maximum sensitivity score	108	108	72	28	108	162	120	120
Average sensitivity score	25.27	27.40	17.08	12.55	29.06	35.00	34.73	31.44

Species' sensitivity scores are calculated as their niche breadth*reliance, with higher values indicating species less sensitive to changes in resource abundance or availability. Equivalent, PECBMS-only *BREAKPOINT* sets for the forest type and region indicators are presented in Table S7. ‘0/1’ identifies species that were interchangeable in any given breakpoint set due to equal sensitivity scores – see specific note for each indicator for further details.

*Species also included in current pan-European forest bird indicator (for full list see http://www.ebcc.info/index.php?ID=459).

a Either species could be included.

b Any one of three could be included.

c Any two of three could be included.

### Additional indicators

The size of the *MINIMAL* set for each of the additional indicators ranged between six (both forest-type indicators) and nine (North region indicator). There was some overlap in the species' composition of these sets, and they were identical for South, West and East indicators, but no species was present in all (Table 1). Again, for each indicator, the average sensitivity score decreased with increasing set size, with an equivalent trend of diminishing gains observed (Fig. 2). The number of species in the *SENSITIVE* set varied between 26 (conifer-dominated forest indicator) and 39 (South region indicator) (Tables S5). Piecewise regression identified *BREAKPOINT* sets for each indicator as: conifer-dominated forest – 12 species, broadleaf-dominated forest – 11 species, North – 16 species, South – 17 species, East – 15 species, West – 16 species (Table 2). Breaks at these points offered significant improvements (P < 0.001) to the basic linear model for that indicator in each case.

Six of the eight species in the *MINIMAL* set of the pan-European indicator are currently covered by PECBMS. When the candidate species' pool was restricted only to those currently covered by PECBMS, a minimum of nine species were required to provide full resource coverage, with *Accipiter gentilis* and *Tetrao urogallus*, the two species from the original indicator not currently covered by PECBMS, replaced by *Accipiter nisus*, *Buteo buteo* and *Parus cristatus* (Table 1). The *SENSITIVE* set for the PECBMS-only indicator contained 30 species, whilst the *BREAKPOINT* set contained 15 species. This set contained nine of the species included in the *BREAKPOINT* set for the full community, which also contained 15 species, but had an average sensitivity score of 27.4 compared to 25.3 for the equivalent set drawn from the full community (Table 2). Restricting species' selection to those currently covered by PECBMS altered the composition of all the additional indicators derived, with the average sensitivity score for any given set size higher (i.e. less sensitive) as a consequence (Fig S1; Tables S6, S7, S8).

**Figure 3:**
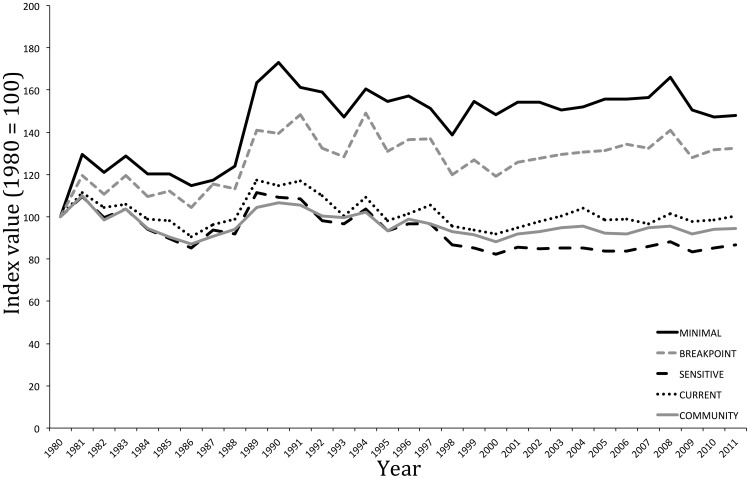
Temporal dynamics of pan-European woodland bird indicator, drawn from species currently covered by PECBMS, between 1980 and 2011. Lines show index values, based on the geometric mean of constituent species' population trends, for *MINIMAL*, *BREAKPOINT*, *SENSITIVE*, the existing pan-European woodland bird index (*CURRENT*) and *COMMUNITY* sets. Equivalent figures for the regional and woodland type indicators are provided in Figures S2, S3, S4, S5, S6, S7.

The temporal trends between 1980 and 2011 of each alternative Pan-European indicator set are shown in Figure 3; equivalent figures for regional and forest-type indicators are provided in the (Figures S2, S3, S4, S5, S6, S7). Results from the comparisons of inter-annual change between alternative species sets for each indicator type and 2011 index values for each are summarised in Table 3. In all cases, except East – *MINIMAL*, South – *MINIMAL* and Broadleaf – *BREAKPOINT*, there was a strong positive correlation between all inter-annual changes in index values and the corresponding *COMMUNITY* index. In the majority of cases with significant correlations, the 95% confidence intervals around their slopes did not encompass one, demonstrating that the scale of inter-annual change of indicator sets was greater than that for the *COMMUNITY* index. In all cases the index value of the *MINIMAL* set was highest and, with the exception of the South indicator, the 2011 community index value fell between that of the *BREAKPOINT* (which was higher) and *SENSITIVE* (which was lower) sets; for the South indicator, the *COMMUNITY* index was very similar to that of the *MINIMAL* index.

**Table 3:** Summary of comparisons between the temporal dynamics of alternative index sets (*MINIMAL*, *BREAKPOINT*, *SENSITIVE* and, for the pan-European and regional indicators, existing indicator sets *CURRENT*) for each indicator type and that of an index based on the population dynamics of all species in the candidate pool from which the sets had been drawn (*COMMUNITY*).

Indicator	Indicator set (Number of species)	Slope (95% CI)	*r*	2011 Index value^a^
Pan-European	*MINIMAL (9)*	0.33 (0.21–0.45)	0.72***	148
	*BREAKPOINT (15)*	0.39 (0.28–0.51)	0.79***	132.5
	*SENSITIVE (30)*	0.74 (0.61–0.89)	0.89***	86.7
	*CURRENT (33)*	0.70 (0.52–0.92)	0.81***	100.5
	*COMMUNITY (58)*			94.3
				
East	*MINIMAL (9)*	0.12 (–0.04–0.29)	0.29*ns*	116.4
	*BREAKPOINT (15)*	0.38 (0.25–0.52)	0.75***	100.1
	*SENSITIVE (31)*	0.60 (0.52–0.68)	0.95***	107.5
	*CURRENT (27)*	0.88 (0.58–1.30)	0.71***	89.5
	*COMMUNITY (52)*			105.4
				
West	*MINIMAL (9)*	0.46 (0.35–0.58)	0.85***	154.2
	*BREAKPOINT (17)*	0.77 (0.61–0.96)	0.86***	89.1
	*SENSITIVE (32)*	0.79 (0.73–0.86)	0.98***	105.3
	*CURRENT (26)*	0.76 (0.65–0.89)	0.93***	108.4
	*COMMUNITY (48)*			102.5
				
North	*MINIMAL (9)*	0.42 (0.27–0.60)	0.70***	77.7
	*BREAKPOINT (16)*	0.59 (0.37–0.88)	0.67***	69.8
	*SENSITIVE (24)*	0.88 (0.82–0.94)	0.98***	72.4
	*CURRENT (26)*	0.99 (0.75–1.29)	0.82***	68.2
	*COMMUNITY (28)*			71.3
				
South	*MINIMAL (9)*	0.29 (–0.23–1.01)	0.25*ns*	102.6
	*BREAKPOINT (18)*	1.00 (0.54–1.85)	0.63*	94.1
	*SENSITIVE (36)*	0.92 (0.79–1.07)	0.95***	94.1
	*CURRENT (24)*	0.94 (0.58–1.49)	0.72***	80.9
	*COMMUNITY (50)*			102.2
				
Broadleaf	*MINIMAL (6)*	0.10 (0.01–0.20)	0.39*	228.8
	*BREAKPOINT (11)*	0.16 (−0.04–0.38)	0.29*ns*	112.5
	*SENSITIVE (25)*	0.63 (0.46–0.85)	0.78***	88.9
	*COMMUNITY (52)*			95.9
				
Conifer	*MINIMAL (7)*	0.36 (0.25–0.48)	0.78***	110.9
	*BREAKPOINT (12)*	0.57 (0.37–0.80)	0.72***	92.2
	*SENSITIVE (22)*	0.75 (0.63–0.89)	0.91***	87.0
	*COMMUNITY (35)*			87.0

Data presented are the slope (95% confidence interval) and correlation coefficient *r* of the relationship between the inter-annual changes of *COMMUNITY* and each alternative indicator set, derived using Type II major axis regression. Slope values less than one reflect greater inter-annual changes in the specific indicator relative to that of *COMMUNITY*. The 2011 index value for each alternative indicator is also shown. *P<0.05; **P<0.01; ***P<0.001; *ns* – not significant.

a Calculated from the geometric mean of constituent species' population change between 1980 (1982 for East and 1989 for South) and 2011 [13].

## Discussion

The smallest indicator set providing full coverage of the resources exploited by the wider community of 80 European forest bird species contained eight species. The average sensitivity across constituent species increased (sensitivity score decreased) with the addition of each extra species to the indicator set, up to a set of 40 species, but piecewise regression identified a breakpoint set at 15 species, beyond which the rate at which sensitivity increased with increasing set size slowed. Exploration of additional indicator sets, designed to represent particular regions and forest types or restricted to species currently covered by PECBMS, revealed a degree of overlap with this main pan-European indicator set, but the differences identified emphasised the need to consider indicator objectives when selecting constituent species.

### Meeting the key attributes of a good indicator

The indicator sets identified in this study correspond well with the key indicator attributes: that an indicator should be representative, reactive, respond predictably and be straightforward to measure, analyse and interpret [9]. Representativeness is highly prioritised here through adherence to the first rule, which stipulates that all resource types exploited by the wider community should be exploited by at least one species in the indicator. Quantitatively ensuring this makes the indicator sets identified unusual amongst ecological indicators developed to date.

A reactive indicator is comprised of the most sensitive species possible because they are more likely to respond to ecological change [17]. Our selection process addresses this requirement by ensuring that the optimal combination identified for each indicator set size comprised the most sensitive species that, between them, offer full coverage of all resource types. We show that, to a point, average sensitivity increases with increasing set size for each indicator type examined, and identify the most sensitive possible combination. However, it is evident that there are diminishing gains in sensitivity with increasing indicator set size and increasing the number of species included beyond the *MINIMAL* set introduces increasing redundancy into the indicator. This trade-off between redundancy and sensitivity is explored in more detail below.

Our species' selection protocol uses an equivalent resource requirements matrix to that used by Wade *et al*. [33] to link the response of forest birds to changes in land-use and management in European forests. This continuity sets our approach within a mechanistic framework linking population dynamics and environmental change, with the resultant indicator therefore expected to respond to changes in resource availability in forest habitats in a predictable way. An equivalent association between risk and population growth rates has been shown for a number of taxa and spatial scales [34], [35], providing further evidence that coarse resource quantification is appropriate in assessing species' responses to ecological change and thus is a suitable basis for indicator species' selection.

The final attribute of a good indicator is that it should be easy to measure, analyse and interpret. Six of the eight species in the *MINIMAL* set for the pan-European indicator are currently covered by PECBMS, with pan-European population data for these species readily available. However, the selection of two species not currently covered by PECBMS identifies a possible gap in this monitoring scheme in terms of forest bird species. Our results suggest that including the ‘missing’ species, *Accipiter gentilis* and *Tetrao urogallus*, in future PECBMS indicators should be seriously considered. These species are monitored by other methods in a number of countries so it might be possible to incorporate these data for analysis and interpretation of the minimal dominating set as a forest bird indicator. The derivation of PECBMS-only indicator sets further supports the suggestion that current coverage does not embrace a fully representative suite of species. Only 58 of the 80 species in the initial candidate species' pool are currently covered and the total niche space occupied by these species is approximately 10% smaller than that of the full community. Restricting the initial species' pool to those currently covered by PECBMS reduced the total niche space occupied by candidate species for all indicator types explored, suggesting that any indicator based on currently covered species would not be fully representative of the wider target community and could be unresponsive to certain changes to forest habitats. In all cases, the “missing” resources (i.e. those used by the wider community but not by PECBMS species) were related to diet rather than nest site and were almost exclusively associated with ground-dwelling vertebrate prey in different habitat types, suggesting that improved monitoring coverage of raptors and owl populations would be beneficial [36].

### Sensitivity versus redundancy

At small indicator set sizes, average sensitivity scores fell sharply with increasing indicator set size as the generalist species (included to ensure full resource coverage in sets with fewer species) were replaced by combinations of more specialised species that, between them, offered the same coverage. For example, the increase from the optimal set of eight species to the optimal set of nine species for the pan-European indicator saw *Columba palumbus* (sensitivity score of 78) replaced by *Pyrrhula pyrrhula* and *Columba oenas* (sensitivity scores of 15 and 30 respectively), with *Columba oenas* then replaced by *Tetrao tetrix* and *Emberiza rustica* (sensitivity scores of 24 and 9 respectively) in the optimal combination of ten species. Inclusion of generalist species in the indicator may be a concern because it reduces the probability of the indicator acting as an early warning system [37]; generalist species are less likely to respond to ecological change because they have greater ability to switch to other resources and a lower proportion of their resources are likely to be affected [38]. However, if a generalist species is uniquely exploiting a particular resource it is important that it is included in the indicator set because, by definition, the indicator is intended to be a good representation of the community as a whole.

For each indicator type, the *SENSITIVE* set, i.e. that with the lowest average sensitivity score, was at least three times larger than the *MINIMAL* set and, in the case of the broadleaf-dominated forest indicator, was 5.5 times the size. However, the rate of increase in sensitivity with increasing number of species declined once the particularly generalist species had been replaced. With the addition of each species to the smallest set, redundancy within the indicator is likely to increase, with a greater number of species exploiting each resource type. Redundancy in an indicator increases the sampling effort required to gather data beyond the minimum required [39], [40] and also increases bias toward those resources that are exploited by multiple species, potentially causing over or under estimation of the impact of ecological changes depending on whether the resources affected are exploited by many or few species. Conversely, a lack of redundancy means the indicator may be sensitive to specific external factors, such as disease or hunting pressure, that affect individual constituent species and that it is not designed to indicate. This susceptibility of small indicator sets to stochastic or species-specific events is demonstrated by the large inter-annual changes (shallower slope) observed for the *MINIMAL* sets compared to that of the other alternative species' sets for each indicator type (Table 3).

The *MINIMAL* and *SENSITIVE* sets, which minimise redundancy and maximise sensitivity respectively, represent the two extremes of the trade-off between these two characteristics. Balancing this trade-off between indicator characteristics is likely to be context dependent [12], according to the preferences or requirements of the end user. Understanding the contexts that benefit sensitivity over redundancy or *vice versa* requires further investigation but for now we would recommend the *BREAKPOINT* set identified by piecewise regression as this identifies the set size at which the rate of increasing sensitivity with an increasing number of species slows and the addition of further species only marginally improves sensitivity. It is important to emphasise that, by identifying a subset of species for inclusion in an aggregate indicator, we are not suggesting that monitoring be restricted solely to these species. Whilst the value of multi-species indicators is demonstrated by their role in environmental management, sustainable development and biodiversity conservation policy and practice [41], we would stress that all species should be monitored or surveyed on a regular basis, whether annually or less frequently, for a host of other reasons, including reporting obligations under the EU Birds Directive.

### Adapting the indicator to different monitoring requirements

The pan-European forest bird indicator corresponds to the scale at which much policy and targeting is set and offers a general picture of the health of forest bird communities. However, conservation and management strategies are often implemented at national or regional scales, so regional indicators could be more appropriate to monitor their effects in more detail. Indeed, PECBMS data have revealed stark differences in population trends of forest birds between regions [4]. Our regional indicators highlighted some differences in the constituent species of forest bird indicators at this scale but the level of overlap may have been lower and even more regionally-focused if species with more limited ranges were included. Some countries, particularly in the North region, have disproportionately large areas but species ranging over large areas were excluded from the regional analyses if this area comprised relatively few countries because the candidate species were all drawn from those used for the main pan-European indicator, which stipulated a range spanning five or more European countries for inclusion. An alternative would be to use a criterion based on area rather than number of countries for inclusion in the candidate species pool for regional indicators. The two forest-type specific indicators also highlight the need to focus species' selection to specific indicator requirements; whilst there was some overlap between constituent species there were subtle differences in composition.

### Assessing indicator characteristics

Varying the species selected for a particular indicator had a significant impact on index value and the subsequent inference drawn about wider community it reflected. Whilst the pattern of temporal dynamics was broadly similar across alternative species' sets for the same indicator type, the extent of inter-annual change varied meaning that absolute index values were markedly different. Thus, for example, an index based on the *MINIMAL* set for the pan-European indicator suggests woodland bird populations are increasing whilst an index based on the *SENSITIVE* set suggests populations have declined by about 13% since 1980; the *COMMUNITY* index suggests actual trends fall somewhere in between these two, with slight population declines of about 6%. *MINIMAL* sets aside, there was broad concordance between index values based on alternative species' sets and the *COMMUNITY* index across indicator types. This provides support both for our approach to selecting suitable indicator species and for the existing multi-species indicators though, for the reasons outlined above, current indicators may not be fully representative of the wider community (i.e. those not covered by PECBMS). We would also expect the objectively selected species' sets to be more ‘future-proof’ than the current sets. Our results show that, on average, European woodland bird populations have remained relatively stable over the past three decades, although individual species have shown marked increases and declines in population (www.ebcc.info/). However, the regional indicators suggest that this overall stability masks population declines in the North, which are offset by increases in other regions. No pan-European forest-type specific indicators currently exist but our *BREAKPOINT* and *SENSITIVE* sets for both broadleaf- and conifer-dominated forest closely align to their respective *COMMUNITY* indicators and we would recommend adopting them more formally. These indicators suggest that, whilst populations of species associated with broadleaf-dominated forests have remained broadly stable, those of species related to conifer-dominated forests have declined by around 10%.

## Conclusions

The majority of ecological indicators developed to date are not selected using objective criteria and may therefore not be truly representative; there is a risk they will not reflect environmental changes affecting resources used by the wider community but not by the indicator species. Our analyses suggest this is true of the current pan-European forest bird indicator, with constituent species not covering the full range of resource types used by the European forest bird community it is intended to represent. We have applied an objective, niche-based framework to species' selection to identify a pan-European forest bird indicator that ensures representativeness and other key indicator attributes are met. By comparing indicator sets drawn from species currently covered by PECBMS and the full forest bird community using this framework, we have identified potential important gaps in coverage and recommend that greater inclusion of raptor and owl species would be particularly beneficial. More generally, we show that optimal species' composition and indicator size will be driven by specific indicator objectives and policy needs, both in terms of the initial pool of candidate species and how key characteristics such as sensitivity and redundancy are traded-off. We believe that adopting this niche-based framework for species' selection supports the objective development of multi-species indicators and, in light of previous applications to link land-use change and population trends [18], [34], [35], that it has good potential to be extended to a range of habitats and taxa.

## Supporting Information

Figure S1 **Relationship between the number of species in the indicator and the average sensitivity score of constituent species in the most sensitive combination for that set size for the pan-European and alternative indicators drawn solely from species currently covered by PECBMS.**
(TIFF)

Figure S2 **Temporal dynamics of the East region woodland bird indicator, drawn from species currently covered by PECBMS, between 1982 and 2011.**
(TIFF)

Figure S3 **Temporal dynamics of the West region woodland bird indicator, drawn from species currently covered by PECBMS, between 1980 and 2011.**
(TIFF)

Figure S4 **Temporal dynamics of the North region woodland bird indicator, drawn from species currently covered by PECBMS, between 1980 and 2011.**
(TIFF)

Figure S5 **Temporal dynamics of the South region woodland bird indicator, drawn from species currently covered by PECBMS, between 1989 and 2011.**
(TIFF)

Figure S6 **Temporal dynamics of the broadleaf-dominated woodland bird indicator, drawn from species currently covered by PECBMS, between 1980 and 2011.**
(TIFF)

Figure S7 **Temporal dynamics of the conifer-dominated woodland bird indicator, drawn from species currently covered by PECBMS, between 1980 and 2011.**
(TIFF)

Table S1 **Matrix of summer foraging resource requirements.**
(DOCX)

Table S2 **Matrix of winter foraging resource requirements.**
(DOCX)

Table S3 **Matrix of nesting resource requirements.**
(DOCX)

Table S4 **Reliance scores used in the calculation of species' sensitivity scores.**
(DOCX)

Table S5 ***SENSITIVE*** **sets for the pan-European, forest-type and regional indicators.**
(DOCX)

Table S6 ***MINIMAL*** **sets for the forest-type and regional indicators drawn solely from species currently covered by PECBMS.**
(DOCX)

Table S7 ***BREAKPOINT*** **sets for the forest-type and regional indicators drawn solely from species currently covered by PECBMS.**
(DOCX)

Table S8 ***SENSITIVE*** **sets for the pan-European, forest-type and regional indicators drawn solely from species currently covered by PECBMS.**
(DOCX)

## References

[1] Bengtsson J, Nilsson SG, Franc A, Menozzi P (2000) Biodiversity, disturbances, ecosystem function and management of European forests. Forest Ecology and Management 132: 39–50.

[2] Forest Europe, UNECE FAO (2011) State of Europe's forests. Status and trends in sustainable forest management in Europe. Oslo: Forest Europe Liaison Unit.

[3] Paillet Y, Berges L, Hjalten J, Odor P, Avon C, et al (2010) Biodiversity Differences between Managed and Unmanaged Forests: Meta-Analysis of Species Richness in Europe. Conservation Biology 24: 101–112. 20121845 10.1111/j.1523-1739.2009.01399.x

[4] Gregory RD, Vorisek P, Van Strien A, Meyling AWG, Jiguet F, et al (2007) Population trends of widespread woodland birds in Europe. Ibis 149: 78–97.

[5] Auld G, Gulbrandsen LH, McDermott CL (2008) Certification schemes and the impacts on forests and forestry. Annual Review of Environment and Resources 33: 187–211.

[6] Pullin AS, Báldi A, CAN OE, Dieterich M, Kati V, et al (2009) Conservation focus on Europe: major conservation policy issues that need to be informed by conservation science. Conservation Biology 23: 818–824. 19627313 10.1111/j.1523-1739.2009.01283.x

[7] Gregory RD, Vořišek P, Noble DG, Van Strien A, Klvaňová A, et al (2008) The generation and use of bird population indicators in Europe. Bird Conservation International 18: S223–S244.

[8] Niemi GJ, McDonald ME (2004) Application of ecological indicators. Annual Review of Ecology, Evolution, and Systematics 35: 89–111.

[9] Dale VH, Beyeler SC (2001) Challenges in the development and use of ecological indicators. Ecological Indicators 1: 3–10.

[10] Sætersdal M, Gjerde I, Blom HH (2005) Indicator species and the problem of spatial inconsistency in nestedness patterns. Biological Conservation 122: 305–316.

[11] Roberge J-M, Angelstam P (2006) Indicator species among resident forest birds - A cross-regional evaluation in northern Europe. Biological Conservation 130: 134–147.

[12] Butler SJ, Freckleton RP, Renwick AR, Norris K (2012) An objective, niche-based approach to indicator species selection. Methods in Ecology and Evolution 3: 317–326.

[13] Gregory RD, van Strien A (2010) Wild Bird Indicators: Using Composite Population Trends of Birds as Measures of Environmental Health. Ornithological Science 9: 3–22.

[14] Bradbury RB, Stoate C, Tallowin JRB (2010) Lowland farmland bird conservation in the context of wider ecosystem service delivery. Journal of Applied Ecology 47: 986–993.

[15] Bryce SA, Hughes RM, Kaufmann PR (2002) Development of a bird integrity index: using bird assemblages as indicators of riparian condition. Environmental Management 30: 294–310. 12105768 10.1007/s00267-002-2702-y

[16] Norris K, Harper N (2004) Extinction processes in hot spots of avian biodiversity and the targeting of pre-emptive conservation action. Proceedings of the Royal Society B: Biological Sciences 271: 123–130. 15058387 10.1098/rspb.2003.2576 PMC1691574

[17] Owens IPF, Bennett PM (2000) Ecological basis of extinction risk in birds: habitat loss versus human persecution and introduced predators. Proceedings of the National Academy of Sciences 97: 12144–12148. 10.1073/pnas.200223397 PMC17308 11005835

[18] Butler SJ, Vickery JA, Norris K (2007) Farmland biodiversity and the footprint of agriculture. Science 315: 381–384. 17234947 10.1126/science.1136607

[19] Tucker GM, Evans MI (1997) Habitats for Birds in Europe: A Conservation Strategy for the Wider Environment. Cambridge, UK: Birdlife International.

[20] Snow DW, Perrins CM (1998) The birds of the Western Palearctic. Oxford, UK: Oxford University Press.

[21] Gregory RD, Van Strien A, Vorisek P, Gmelig Meyling AW, Noble DG, et al (2005) Developing indicators for European birds. Philosophical Transactions of the Royal Society B: Biological Sciences 360: 269–288. 10.1098/rstb.2004.1602 PMC1569455 15814345

[22] Fuller RJ, Smith KW, Grice PV, Currie FA, Quine CP (2007) Habitat change and woodland birds in Britain: implications for management and future research. Ibis 149: 261–268.

[23] Tellería JL, Baquero R, Santos T (2003) Effects of forest fragmentation on European birds: implications of regional differences in species richness. Journal of Biogeography 30: 621–628.

[24] Angelstam P, Roberge JM, Lohmus A, Bergmanis M, Brazaitis G, et al (2004) Habitat modelling as a tool for landscape-scale conservation–a review of parameters for focal forest birds. Ecological Bulletins 51: 427–453.

[25] Mikusiński G, Gromadzki M, Chylarecki P (2001) Woodpeckers as Indicators of Forest Bird Diversity. Conservation Biology 15: 208–217.

[26] Thaxter CB, Joys AC, Gregory RD, Baillie SR, Noble DG (2010) Hypotheses to explain patterns of population change among breeding bird species in England. Biological Conservation 143: 2006–2019.

[27] Gil-Tena A, Brotons L, Saura S (2009) Mediterranean forest dynamics and forest bird distribution changes in the late 20th century. Global Change Biology 15: 474–485.

[28] Fomin FV, Grandoni F, Pyatkin AV, Stepanov AA (2008) Combinatorial bounds via measure and conquer: Bounding minimal dominating sets and applications. ACM Trans Algorithms 5: 1–17.

[29] Couturier J-F, Heggernes P, Hof P, Kratsch D (2012) Minimal Dominating Sets in Graph Classes: Combinatorial Bounds and Enumeration. In: Bieliková M, Friedrich G, Gottlob G, Katzenbeisser S, Turán G, editors. SOFSEM 2012: Theory and Practice of Computer Science: Springer Berlin Heidelberg. pp. 202–213.

[30] Gregory RD, Noble D, Field RH, Marchant JH, Raven M, et al (2003) Using birds as indicators of biodiversity. Ornis Hungarica 12–13: 11–24.

[31] Legendre P (2008) lmodel2: Model II Regression. Available: http://www.cran.r-project.org

[32] Legendre P, Legendre L (1998) Numerical Ecology. Amsterdam: Elsevier.

[33] Wade ASI, Barov B, Burfield IJ, Gregory RD, Norris K, et al (2013) Quantifying the detrimental impacts of land-use and management change on European forest bird populations. PLoS One 8: e64552. 23704997 10.1371/journal.pone.0064552 PMC3660351

[34] Butler SJ, Brooks D, Feber RE, Storkey J, Vickery JA, et al (2009) A cross-taxonomic index for quantifying the health of farmland biodiversity. Journal of Applied Ecology 46: 1154–1162.

[35] Butler SJ, Boccaccio L, Gregory RD, Vorisek P, Norris K (2010) Quantifying the impact of land-use change to European farmland bird populations. Agriculture Ecosystems & Environment 137: 348–357.

[36] Kovaács A, Mammen UCC, Wernham CV (2008) European Monitoring for Raptors and Owls: State of the Art and Future Needs. AMBIO: A Journal of the Human Environment 37: 408–412. 10.1579/0044-7447(2008)37[408:emfrao]2.0.co;2 18833792

[37] Carignan V, Villard MA (2002) Selecting indicator species to monitor ecological integrity: a review. Environmental Monitoring and Assessment 78: 45–61. 12197640 10.1023/a:1016136723584

[38] Norris K, Harper N (2004) Extinction processes in hot spots of avian biodiversity and the targeting of pre–emptive conservation action. Proceedings of the Royal Society of London Series B: Biological Sciences 271: 123–130. 15058387 10.1098/rspb.2003.2576 PMC1691574

[39] Bladt J, Larsen FW, Rahbek C (2008) Does taxonomic diversity in indicator groups influence their effectiveness in identifying priority areas for species conservation? Animal Conservation 11: 546–554.

[40] Lewandowski AS, Noss RF, Parsons DR (2010) The effectiveness of surrogate taxa for the representation of biodiversity. Conservation Biology 24: 1367–1377. 20455907 10.1111/j.1523-1739.2010.01513.x

[41] Niemeijer D, de Groot RS (2008) A conceptual framework for selecting environmental indicator sets. Ecological Indicators 8: 14–25.

