# Supplementary material for: A Niche-Based Framework to Assess Current Monitoring of European Forest Birds and Guide Indicator Species' Selection
Source: PLoS One. 2014 May 12;9(5):e97217. doi: 10.1371/journal.pone.0097217 (PMC4018337; doi:10.1371/journal.pone.0097217)
Supplement: Table S2 — Matrix of winter foraging resource requirements. (DOCX) [file pone.0097217.s009.docx]

**Table S2**: Winter diet and foraging resource use for the 80 candidate species. Each possible combination of diet, forest type, horizontal and vertical habitat for each species was identified and regarded as one niche type in analyses.

|  | Diet | | | | |  | Forest type | | |  | Horizontal habitat | |  | Vertical habitat | | |
| --- | --- | --- | --- | --- | --- | --- | --- | --- | --- | --- | --- | --- | --- | --- | --- | --- |
| Species | Below-ground inverts | Above-ground inverts | Plant material | Seeds | Vertebrates |  | Deciduous | Conifer | Mixed |  | Edge | Core |  | Ground | Shrub | Canopy |
| *Ciconia nigra* | 0 | 0 | 0 | 0 | 0 |  | 0 | 0 | 0 |  | 0 | 0 |  | 0 | 0 | 0 |
| *Pernis apivorus* | 0 | 0 | 0 | 0 | 0 |  | 0 | 0 | 0 |  | 0 | 0 |  | 0 | 0 | 0 |
| *Accipiter gentilis* | 0 | 0 | 0 | 0 | 1 |  | 1 | 1 | 1 |  | 1 | 1 |  | 1 | 1 | 1 |
| *Accipiter nisus* | 0 | 0 | 0 | 0 | 1 |  | 0 | 1 | 1 |  | 1 | 1 |  | 0 | 1 | 1 |
| *Buteo buteo* | 1 | 1 | 0 | 0 | 1 |  | 1 | 0 | 0 |  | 1 | 0 |  | 1 | 0 | 0 |
| *Aquila pomarina* | 0 | 0 | 0 | 0 | 0 |  | 0 | 0 | 0 |  | 0 | 0 |  | 0 | 0 | 0 |
| *Hieraaetus pennatus* | 0 | 0 | 0 | 0 | 0 |  | 0 | 0 | 0 |  | 0 | 0 |  | 0 | 0 | 0 |
| *Bonasa bonasia* | 0 | 0 | 1 | 1 | 0 |  | 0 | 1 | 1 |  | 1 | 0 |  | 0 | 1 | 1 |
| *Tetrao tetrix* | 0 | 0 | 1 | 0 | 0 |  | 1 | 1 | 1 |  | 1 | 0 |  | 0 | 1 | 1 |
| *Tetrao urogallus* | 0 | 0 | 1 | 0 | 0 |  | 0 | 1 | 0 |  | 1 | 1 |  | 0 | 0 | 1 |
| *Scolopax rusticola* | 1 | 1 | 0 | 0 | 0 |  | 1 | 1 | 1 |  | 1 | 0 |  | 1 | 0 | 0 |
| *Columba oenas* | 0 | 0 | 1 | 1 | 0 |  | 1 | 1 | 1 |  | 1 | 0 |  | 1 | 0 | 0 |
| *Columba palumbus* | 0 | 0 | 1 | 1 | 0 |  | 1 | 1 | 1 |  | 1 | 0 |  | 1 | 1 | 1 |
| *Cuculus canorus* | 0 | 0 | 0 | 0 | 0 |  | 0 | 0 | 0 |  | 0 | 0 |  | 0 | 0 | 0 |
| *Bubo bubo* | 0 | 0 | 0 | 0 | 1 |  | 1 | 1 | 1 |  | 1 | 0 |  | 1 | 0 | 0 |
| *Glaucidium passerinum* | 0 | 0 | 0 | 0 | 1 |  | 1 | 1 | 1 |  | 1 | 0 |  | 1 | 1 | 1 |
| *Strix aluco* | 0 | 0 | 0 | 0 | 1 |  | 1 | 1 | 1 |  | 1 | 1 |  | 1 | 1 | 1 |
| *Strix uralensis* | 0 | 0 | 0 | 0 | 1 |  | 1 | 1 | 1 |  | 1 | 0 |  | 1 | 1 | 1 |
| *Aegolius funereus* | 0 | 0 | 0 | 0 | 1 |  | 0 | 1 | 1 |  | 1 | 1 |  | 1 | 1 | 1 |
| *Caprimulgus europaeus* | 0 | 0 | 0 | 0 | 0 |  | 0 | 0 | 0 |  | 0 | 0 |  | 0 | 0 | 0 |
| *Jynx torquilla* | 0 | 0 | 0 | 0 | 0 |  | 0 | 0 | 0 |  | 0 | 0 |  | 0 | 0 | 0 |
| *Picus canus* | 1 | 1 | 0 | 0 | 0 |  | 1 | 0 | 1 |  | 1 | 1 |  | 1 | 0 | 0 |
| *Picus viridis* | 1 | 1 | 0 | 0 | 0 |  | 1 | 0 | 1 |  | 1 | 0 |  | 1 | 0 | 0 |
| *Dryocopus martius* | 0 | 1 | 0 | 0 | 0 |  | 1 | 1 | 1 |  | 1 | 1 |  | 1 | 0 | 1 |
| *Dendrocopos major* | 0 | 1 | 0 | 1 | 0 |  | 1 | 1 | 1 |  | 1 | 1 |  | 0 | 0 | 1 |
| *Dendrocopos medius* | 0 | 1 | 0 | 0 | 0 |  | 1 | 0 | 0 |  | 0 | 1 |  | 0 | 0 | 1 |
| *Dendrocopos leucotos* | 0 | 1 | 0 | 0 | 0 |  | 1 | 0 | 1 |  | 1 | 1 |  | 0 | 0 | 1 |
| *Dendrocopos minor* | 0 | 1 | 0 | 0 | 0 |  | 1 | 0 | 1 |  | 1 | 0 |  | 0 | 1 | 1 |
| *Picoides tridactylus* | 0 | 1 | 0 | 0 | 0 |  | 0 | 1 | 1 |  | 0 | 1 |  | 0 | 0 | 1 |
| *Lullula arborea* | 0 | 0 | 0 | 1 | 0 |  | 1 | 1 | 1 |  | 1 | 0 |  | 1 | 1 | 0 |
| *Anthus trivialis* | 0 | 0 | 0 | 0 | 0 |  | 0 | 0 | 0 |  | 0 | 0 |  | 0 | 0 | 0 |
| *Troglodytes troglodytes* | 0 | 1 | 0 | 0 | 0 |  | 1 | 1 | 1 |  | 1 | 1 |  | 1 | 1 | 0 |
| *Prunella modularis* | 0 | 1 | 0 | 1 | 0 |  | 1 | 1 | 1 |  | 1 | 0 |  | 1 | 1 | 0 |
| *Erithacus rubecula* | 0 | 1 | 1 | 1 | 0 |  | 1 | 1 | 1 |  | 1 | 0 |  | 1 | 0 | 0 |
| *Luscinia megarhynchos* | 0 | 0 | 0 | 0 | 0 |  | 0 | 0 | 0 |  | 0 | 0 |  | 0 | 0 | 0 |
| *Phoenicurus phoenicurus* | 0 | 0 | 0 | 0 | 0 |  | 0 | 0 | 0 |  | 0 | 0 |  | 0 | 0 | 0 |
| *Turdus merula* | 1 | 1 | 1 | 0 | 0 |  | 1 | 1 | 1 |  | 1 | 1 |  | 1 | 1 | 0 |
| *Turdus philomelos* | 1 | 1 | 1 | 0 | 0 |  | 1 | 1 | 1 |  | 1 | 0 |  | 1 | 1 | 0 |
| *Turdus iliacus* | 1 | 1 | 1 | 0 | 0 |  | 1 | 0 | 1 |  | 1 | 0 |  | 1 | 1 | 1 |
| *Turdus viscivorus* | 1 | 1 | 1 | 0 | 0 |  | 1 | 1 | 1 |  | 1 | 0 |  | 1 | 1 | 1 |
| *Locustella fluviatilis* | 0 | 0 | 0 | 0 | 0 |  | 0 | 0 | 0 |  | 0 | 0 |  | 0 | 0 | 0 |
| *Hippolais icterina* | 0 | 0 | 0 | 0 | 0 |  | 0 | 0 | 0 |  | 0 | 0 |  | 0 | 0 | 0 |
| *Hippolais polyglotta* | 0 | 0 | 0 | 0 | 0 |  | 0 | 0 | 0 |  | 0 | 0 |  | 0 | 0 | 0 |
| *Sylvia borin* | 0 | 0 | 0 | 0 | 0 |  | 0 | 0 | 0 |  | 0 | 0 |  | 0 | 0 | 0 |
| *Sylvia atricapilla* | 0 | 0 | 1 | 0 | 0 |  | 1 | 0 | 1 |  | 1 | 1 |  | 1 | 1 | 1 |
| *Phylloscopus trochiloides* | 0 | 0 | 0 | 0 | 0 |  | 0 | 0 | 0 |  | 0 | 0 |  | 0 | 0 | 0 |
| *Phylloscopus bonelli* | 0 | 0 | 0 | 0 | 0 |  | 0 | 0 | 0 |  | 0 | 0 |  | 0 | 0 | 0 |
| *Phylloscopus sibilatrix* | 0 | 0 | 0 | 0 | 0 |  | 0 | 0 | 0 |  | 0 | 0 |  | 0 | 0 | 0 |
| *Phylloscopus collybita* | 0 | 1 | 1 | 0 | 0 |  | 1 | 1 | 1 |  | 1 | 0 |  | 0 | 1 | 1 |
| *Phylloscopus trochilus* | 0 | 0 | 0 | 0 | 0 |  | 0 | 0 | 0 |  | 0 | 0 |  | 0 | 0 | 0 |
| *Regulus regulus* | 0 | 1 | 0 | 0 | 0 |  | 1 | 1 | 1 |  | 1 | 1 |  | 0 | 1 | 1 |
| *Regulus ignicapilla* | 0 | 1 | 0 | 0 | 0 |  | 0 | 1 | 1 |  | 1 | 0 |  | 0 | 1 | 1 |
| *Muscicapa striata* | 0 | 0 | 0 | 0 | 0 |  | 0 | 0 | 0 |  | 0 | 0 |  | 0 | 0 | 0 |
| *Ficedula parva* | 0 | 0 | 0 | 0 | 0 |  | 0 | 0 | 0 |  | 0 | 0 |  | 0 | 0 | 0 |
| *Ficedula albicollis* | 0 | 0 | 0 | 0 | 0 |  | 0 | 0 | 0 |  | 0 | 0 |  | 0 | 0 | 0 |
| *Ficedula hypoleuca* | 0 | 0 | 0 | 0 | 0 |  | 0 | 0 | 0 |  | 0 | 0 |  | 0 | 0 | 0 |
| *Aegithalos caudatus* | 0 | 1 | 0 | 0 | 0 |  | 1 | 0 | 1 |  | 1 | 1 |  | 0 | 1 | 1 |
| *Parus palustris* | 0 | 1 | 1 | 1 | 0 |  | 1 | 0 | 1 |  | 1 | 1 |  | 1 | 1 | 1 |
| *Parus lugubris* | 0 | 1 | 0 | 0 | 0 |  | 1 | 0 | 1 |  | 1 | 1 |  | 1 | 1 | 1 |
| *Parus montanus* | 0 | 0 | 0 | 1 | 0 |  | 1 | 1 | 1 |  | 1 | 1 |  | 0 | 1 | 1 |
| *Parus cristatus* | 0 | 1 | 1 | 1 | 0 |  | 0 | 1 | 0 |  | 0 | 1 |  | 1 | 1 | 1 |
| *Parus ater* | 0 | 1 | 0 | 1 | 0 |  | 0 | 1 | 0 |  | 1 | 1 |  | 0 | 0 | 1 |
| *Parus caeruleus* | 0 | 1 | 1 | 1 | 0 |  | 1 | 0 | 0 |  | 1 | 1 |  | 0 | 1 | 1 |
| *Parus major* | 0 | 1 | 1 | 1 | 0 |  | 1 | 0 | 1 |  | 1 | 1 |  | 1 | 1 | 1 |
| *Sitta europaea* | 0 | 1 | 0 | 1 | 0 |  | 1 | 0 | 1 |  | 1 | 1 |  | 0 | 0 | 1 |
| *Certhia familiaris* | 0 | 1 | 0 | 0 | 0 |  | 0 | 1 | 1 |  | 1 | 1 |  | 0 | 0 | 1 |
| *Certhia brachydactyla* | 0 | 1 | 0 | 0 | 0 |  | 1 | 0 | 1 |  | 1 | 1 |  | 0 | 0 | 1 |
| *Oriolus oriolus* | 0 | 0 | 0 | 0 | 0 |  | 0 | 0 | 0 |  | 0 | 0 |  | 0 | 0 | 0 |
| *Garrulus glandarius* | 0 | 1 | 1 | 1 | 0 |  | 1 | 0 | 1 |  | 0 | 1 |  | 1 | 0 | 1 |
| *Nucifraga caryocatactes* | 0 | 0 | 0 | 1 | 0 |  | 0 | 1 | 0 |  | 1 | 1 |  | 1 | 1 | 1 |
| *Fringilla coelebs* | 0 | 0 | 1 | 1 | 0 |  | 1 | 1 | 1 |  | 1 | 1 |  | 1 | 0 | 0 |
| *Serinus serinus* | 0 | 0 | 1 | 1 | 0 |  | 0 | 1 | 1 |  | 1 | 0 |  | 1 | 0 | 0 |
| *Carduelis spinus* | 0 | 0 | 0 | 1 | 0 |  | 1 | 1 | 1 |  | 1 | 1 |  | 0 | 0 | 1 |
| *Carduelis flammea* | 0 | 0 | 0 | 1 | 0 |  | 1 | 1 | 1 |  | 1 | 0 |  | 1 | 0 | 1 |
| *Loxia curvirostra* | 0 | 0 | 0 | 1 | 0 |  | 0 | 1 | 0 |  | 1 | 1 |  | 0 | 0 | 1 |
| *Loxia pytyopsittacus* | 0 | 0 | 0 | 1 | 0 |  | 0 | 1 | 0 |  | 1 | 1 |  | 0 | 0 | 1 |
| *Carduelis chloris* | 0 | 0 | 0 | 1 | 0 |  | 1 | 1 | 1 |  | 1 | 0 |  | 1 | 1 | 1 |
| *Pyrrhula pyrrhula* | 0 | 0 | 1 | 1 | 0 |  | 1 | 1 | 1 |  | 1 | 0 |  | 0 | 1 | 0 |
| *C. coccothraustes** | 0 | 0 | 1 | 1 | 0 |  | 1 | 0 | 1 |  | 1 | 1 |  | 1 | 1 | 1 |
| *Emberiza rustica* | 0 | 0 | 0 | 0 | 0 |  | 0 | 0 | 0 |  | 0 | 0 |  | 0 | 0 | 0 |

**Coccothraustes coccothraustes*
