# Supplementary material for: A Niche-Based Framework to Assess Current Monitoring of European Forest Birds and Guide Indicator Species' Selection
Source: PLoS One. 2014 May 12;9(5):e97217. doi: 10.1371/journal.pone.0097217 (PMC4018337; doi:10.1371/journal.pone.0097217)
Supplement: Table S4 — Reliance scores used in the calculation of species' sensitivity scores. (DOCX) [file pone.0097217.s011.docx]

**Table S4.** Reliance scores for each species used in the calculation of species’ sensitivity during the selection of each indicator set. Note reliance scores for the same species may vary between indicators – see main text for details.

|  | Indicator set | | | | | | |
| --- | --- | --- | --- | --- | --- | --- | --- |
| Species | Full | Deciduous | Coniferous | North | South | East | West |
| *Accipiter gentilis* | 1 | 2 | 2 | 1 | 1 | 1 | 1 |
| *Aegithalos caudatus* | 1 | 2 | 2 |  | 1 | 2 | 2 |
| *Aegolius funereus* | 1 |  | 1 | 1 | 1 | 1 | 1 |
| *Aquila pomarina* | 1 | 2 | 2 |  |  | 2 |  |
| *Bonasa bonasia* | 1 |  | 1 | 1 |  | 1 |  |
| *Carduelis spinus* | 1 | 2 | 2 | 1 |  | 1 | 1 |
| *Certhia familiaris* | 1 | 2 | 2 | 1 | 1 | 1 | 1 |
| *Ciconia nigra* | 1 | 2 | 2 |  | 1 | 1 | 1 |
| *C. coccothraustes** | 1 | 1 |  |  | 1 | 2 | 1 |
| *Dendrocopos leucotos* | 1 | 1 |  | 1 | 1 | 1 | 1 |
| *Dendrocopos major* | 1 | 2 | 2 | 1 | 2 | 2 | 1 |
| *Dendrocopos medius* | 1 | 1 |  |  | 1 | 2 | 1 |
| *Dendrocopos minor* | 1 | 1 |  |  | 2 | 2 | 1 |
| *Dryocopus martius* | 1 | 2 | 2 | 1 | 1 | 1 | 1 |
| *Emberiza rustica* | 1 | 2 | 2 | 1 |  |  |  |
| *Ficedula albicollis* | 1 | 1 |  |  |  | 2 |  |
| *Ficedula hypoleuca* | 1 | 1 |  |  | 1 | 2 | 1 |
| *Ficedula parva* | 1 | 2 | 2 |  |  | 1 |  |
| *Garrulus glandarius* | 1 | 1 |  | 1 | 1 | 2 | 1 |
| *Glaucidium passerinum* | 1 | 2 | 2 | 1 |  | 1 | 1 |
| *Hieraaetus pennatus* | 1 | 2 | 2 |  | 2 |  |  |
| *Hippolais icterina* | 1 | 1 |  | 1 |  | 3 | 2 |
| *Loxia curvirostra* | 1 |  | 1 | 1 | 1 | 1 | 1 |
| *Loxia pytyopsittacus* | 1 |  | 1 | 1 |  |  |  |
| *Nucifraga caryocatactes* | 1 |  | 1 | 1 | 1 | 1 | 1 |
| *Oriolus oriolus* | 1 | 1 |  |  | 2 | 2 | 1 |
| *Parus ater* | 1 |  | 1 | 1 | 1 | 1 | 1 |
| *Parus cristatus* | 1 |  | 1 | 1 | 1 | 1 | 1 |
| *Parus lugubris* | 1 | 1 |  |  |  |  |  |
| *Parus montanus* | 1 | 2 | 2 | 1 | 1 | 1 | 1 |
| *Parus palustris* | 1 | 1 |  | 1 | 1 | 2 | 1 |
| *Pernis apivorus* | 1 | 2 | 2 | 1 | 1 | 1 | 1 |
| *Phylloscopus bonelli* | 1 | 2 | 2 |  | 1 |  |  |
| *Phylloscopus sibilatrix* | 1 | 2 | 2 | 1 | 1 | 1 | 1 |
| *Phylloscopus trochiloides* | 1 | 2 | 2 | 1 |  | 1 |  |
| *Picoides tridactylus* | 1 |  | 1 | 1 |  | 1 | 1 |
| *Picus canus* | 1 | 1 |  | 1 | 1 | 1 | 2 |
| *Pyrrhula pyrrhula* | 1 | 2 | 2 | 1 | 1 | 1 | 2 |
| *Regulus ignicapilla* | 1 |  | 1 |  | 1 | 1 | 1 |
| *Regulus regulus* | 1 | 2 | 2 | 1 | 1 | 1 | 1 |
| *Scolopax rusticola* | 1 | 2 | 2 | 1 |  | 1 |  |
| *Sitta europaea* | 1 | 1 |  |  | 1 | 2 | 1 |
| *Strix uralensis* | 1 | 2 | 2 | 1 |  | 1 |  |
| *Tetrao urogallus* | 1 |  | 1 | 1 |  |  |  |
| *Troglodytes troglodytes* | 1 | 2 | 2 |  | 2 | 2 | 2 |
| *Turdus philomelos* | 1 | 2 | 2 | 1 | 2 | 2 | 2 |
| *Turdus viscivorus* | 1 | 2 | 2 | 1 | 2 | 1 | 2 |
| *Accipiter nisus* | 2 | 4 | 4 | 1 | 2 | 2 | 2 |
| *Anthus trivialis* | 2 | 4 | 4 |  | 2 | 2 | 2 |
| *Buteo buteo* | 2 | 2 |  |  | 2 | 2 | 2 |
| *Caprimulgus europaeus* | 2 | 4 | 4 |  | 2 | 2 |  |
| *Carduelis flammea* | 2 | 4 | 4 | 2 |  |  |  |
| *Certhia brachydactyla* | 2 | 2 |  |  | 1 |  | 2 |
| *Columba oenas* | 2 | 4 | 4 |  | 2 | 1 | 2 |
| *Columba palumbus* | 2 | 4 | 4 | 2 | 2 | 2 | 2 |
| *Cuculus canorus* | 2 | 4 | 4 | 2 | 3 | 3 | 3 |
| *Erithacus rubecula* | 2 | 4 | 4 | 2 | 2 | 2 | 2 |
| *Fringilla coelebs* | 2 | 4 | 4 | 2 | 3 | 2 | 2 |
| *Lullula arborea* | 2 | 4 | 4 |  | 2 | 2 |  |
| *Luscinia megarhynchos* | 2 | 2 |  |  | 2 | 3 |  |
| *Muscicapa striata* | 2 | 2 |  | 1 | 2 | 2 | 2 |
| *Parus caeruleus* | 2 | 2 |  |  | 1 | 2 | 2 |
| *Parus major* | 2 | 2 |  |  | 2 | 2 | 2 |
| *Phoenicurus phoenicurus* | 2 | 2 |  |  | 2 | 2 | 2 |
| *Phylloscopus collybita* | 2 | 4 | 4 |  | 2 | 2 | 2 |
| *Phylloscopus trochilus* | 2 | 4 | 4 |  | 1 | 2 | 2 |
| *Picus viridis* | 2 | 2 |  |  | 2 |  | 2 |
| *Prunella modularis* | 2 | 4 | 4 |  | 3 | 2 | 2 |
| *Strix aluco* | 2 | 4 | 4 |  | 1 | 2 | 1 |
| *Sylvia atricapilla* | 2 | 2 |  |  | 2 | 2 | 2 |
| *Sylvia borin* | 2 | 2 |  | 1 | 2 | 2 | 2 |
| *Tetrao tetrix* | 2 | 4 | 4 |  |  |  |  |
| *Turdus merula* | 2 | 4 | 4 | 2 | 3 | 2 | 2 |
| *Bubo bubo* | 3 | 6 | 6 |  | 3 | 2 | 3 |
| *Carduelis chloris* | 3 | 6 | 6 |  | 3 | 3 | 3 |
| *Hippolais polyglotta* | 3 | 3 |  |  | 3 |  |  |
| *Jynx torquilla* | 3 | 3 |  |  | 3 | 3 | 2 |
| *Locustella fluviatilis* | 3 | 6 | 6 |  |  | 3 |  |
| *Serinus serinus* | 3 |  | 3 |  | 3 |  |  |
| *Turdus iliacus* | 3 | 3 |  |  |  | 2 |  |

**Coccothraustes coccothraustes*
