# Supplementary material for: A Niche-Based Framework to Assess Current Monitoring of European Forest Birds and Guide Indicator Species' Selection
Source: PLoS One. 2014 May 12;9(5):e97217. doi: 10.1371/journal.pone.0097217 (PMC4018337; doi:10.1371/journal.pone.0097217)
Supplement: Table S5 — SENSITIVE sets for the pan-European, forest-type and regional indicators. (DOCX) [file pone.0097217.s012.docx]

**Table S5**: Species included in the *SENSITIVE* sets for the main pan-European, forest-type specific and regional indicators. Species’ sensitivity scores are calculated as their niche breadth*reliance, with higher values indicating species less sensitive to changes in resource abundance or availability. ‘0/1’ identifies species that were interchangeable in any given breakpoint set due to equal sensitivity scores.

| Species | Main | Conifer-dominated | Broadleaf-dominated | North | South | East | West |
| --- | --- | --- | --- | --- | --- | --- | --- |
| *Accipiter gentilis* | 1 |  | 1 | 1 | 1 | 1 | 1 |
| *Aquila pomarina* | 1 | 1 | 1 |  |  | 1 |  |
| *Bonasa bonasia** | 1 | 1 |  | 1 |  | 1 |  |
| *Buteo buteo* | 1 |  |  |  | 1 | 1 | 1 |
| *Caprimulgus europaeus* | 1 | 1 | 1 |  | 1 | 1 |  |
| *Ciconia nigra* | 1 | 1 | 1 |  | 1 | 1 | 1 |
| *C. coccothraustes* **^§^***** | 1 |  | 1 |  | 1 | 1 | 1 |
| *Dendrocopos leucotos* | 1 |  | 1 | 1 | 1 | 1 | 1 |
| *Dendrocopos major* | 1 | 1 |  | 1 | 1 | 1 | 1 |
| *Dendrocopos medius** | 1 |  | 1 |  | 1 | 1 | 1 |
| *Dendrocopos minor** | 1 |  | 1 |  | 1 | 1 | 1 |
| *Emberiza rustica** | 1 | 1 | 1 | 1 |  |  |  |
| *Ficedula albicollis** | 1 |  | 1 |  |  | 1 |  |
| *Ficedula hypoleuca** | 1 |  | 1 |  | 1 | 1 | 1 |
| *Ficedula parva* | 1 | 1 | 1 |  |  | 1 |  |
| *Hieraaetus pennatus* | 1 | 1 | 1 |  |  |  |  |
| *Hippolais icterina* | 1 |  | 1 | 1 |  |  |  |
| *Jynx torquilla* | 1 |  | 1 |  | 1 | 1 | 1 |
| *Loxia curvirostra* | 1 | 1 |  | 1 | 1 | 1 | 1 |
| *Loxia pytyopsittacus* | 1 | 1 |  | 1 |  |  |  |
| *Luscinia megarhynchos* | 1 |  | 1 |  | 1 | 1 |  |
| *Muscicapa striata* | 1 |  | 1 | 1 | 1 | 1 | 1 |
| *Nucifraga caryocatactes** | 1 | 1 |  | 1 | 1 | 1 | 1 |
| *Oriolus oriolus* | 1 |  | 1 |  | 1 | 1 | 1 |
| *Parus ater** | 1 | 1 |  | 1 | 1 | 1 | 1 |
| *Parus cristatus** | 1 |  |  | 1 | 1 | 1 | 1 |
| *Pernis apivorus* | 1 | 1 | 1 | 1 | 1 | 1 | 1 |
| *Phylloscopus bonelli** | 1 | 1 | 1 |  | 1 |  |  |
| *Phylloscopus sibilatrix** | 1 | 1 | 1 | 1 | 1 | 1 | 1 |
| *Phylloscopus trochiloides* | 1 | 1 |  | 1 |  | 1 |  |
| *Picoides tridactylus* | 1 | 1 |  | 1 |  | 1 | 1 |
| *Pyrrhula pyrrhula** | 1 | 1 | 1 | 1 | 1 | 1 | 1 |
| *Regulus ignicapilla** | 1 | 1 |  |  | 1 | 1 | 1 |
| *Regulus regulus** | 1 |  | 1 | 1 | 1 | 1 | 1 |
| *Sitta europaea** | 1 |  | 1 |  | 1 |  | 1 |
| *Sylvia borin* | 1 |  | 1 | 1 | 1 | 1 | 1 |
| *Tetrao tetrix* | 1 |  | 0/1^a^ |  |  |  |  |
| *Tetrao urogallus* | 1 | 1 |  | 1 |  |  |  |
| *Troglodytes troglodytes* | 1 | 1 |  |  | 1 | 1 | 1 |
| *Turdus merula* | 1 | 1 |  | 1 | 1 | 1 | 1 |
| *Phoenicurus phoenicurus* |  |  | 0/1^a^ |  |  |  |  |
| *Aegithalos caudatus* |  | 1 |  |  | 1 |  |  |
| *Aegolius funereus* |  | 1 |  |  |  |  |  |
| *Anthus trivialis** |  | 1 |  |  | 1 | 1 | 1 |
| *Scolopax rusticola* |  | 1 |  | 1 |  | 1 |  |
| *Accipiter nisus** |  |  | 1 | 1 |  |  |  |
| *Carduelis spinus** |  |  | 1 | 1 |  | 1 | 1 |
| *Certhia familiaris** |  |  | 1 | 1 | 1 | 1 | 1 |
| *Garrulus glandarius** |  |  | 1 | 1 | 1 |  | 1 |
| *Glaucidium passerinum* |  |  | 1 | 1 |  | 1 | 1 |
| *Parus palustris** |  |  | 1 |  |  |  |  |
| *Picus canus** |  |  | 1 |  |  |  |  |
| *Carduelis flammea* |  |  |  | 1 |  |  |  |
| *Cuculus canorus* |  |  |  | 1 |  |  |  |
| *Parus montanus** |  |  |  | 1 |  |  |  |
| *Turdus philomelos* |  |  |  | 1 |  |  |  |
| *Turdus viscivorus** |  |  |  | 1 |  |  |  |
| *Certhia brachydactyla* |  |  |  |  | 1 |  |  |
| *Columba oenas* |  |  |  |  | 1 | 1 | 1 |
| *Hippolais polyglotta* |  |  |  |  | 1 |  |  |
| *Parus caeruleus* |  |  |  |  | 1 |  |  |
| *Phylloscopus collybita** |  |  |  |  | 1 |  | 1 |
| *Phylloscopus trochilus* |  |  |  |  | 1 |  |  |
| *Picus viridis* |  |  |  |  | 1 |  | 1 |
| Number of species | 40 | 26 | 33 | 32 | 39 | 38 | 34 |
| Average sensitivity score | 16.40 | 12.46 | 8.39 | 22.47 | 23.54 | 22.21 | 22.56 |

**^§^***Coccothraustes coccothraustes*

**Species also included in current pan-European forest bird indicator (for full list see* <http://www.ebcc.info/index.php?ID=459>)

^a^Either species could be included
