# Supplementary material for: A Niche-Based Framework to Assess Current Monitoring of European Forest Birds and Guide Indicator Species' Selection
Source: PLoS One. 2014 May 12;9(5):e97217. doi: 10.1371/journal.pone.0097217 (PMC4018337; doi:10.1371/journal.pone.0097217)
Supplement: Table S6 — MINIMAL sets for the forest-type and regional indicators drawn solely from species currently covered by PECBMS. (DOCX) [file pone.0097217.s013.docx]

**Table S6**: Species included in the *MINIMAL* sets drawn only from species currently covered by PECBMS for forest-type specific and regional indicators. Species’ sensitivity scores are calculated as their niche breadth*reliance, with higher values indicating species less sensitive to changes in resource abundance or availability.

| Species | Conifer-dominated | Broadleaf-dominated | North | South | East | West |
| --- | --- | --- | --- | --- | --- | --- |
| *Accipiter nisus* | 1 |  | 1 | 1 | 1 | 1 |
| *Bonasa bonasia* | 1 |  |  |  |  |  |
| *Dendrocopos major* | 1 |  | 1 | 1 | 1 | 1 |
| *Nucifraga caryocatactes* | 1 |  | 1 | 1 | 1 | 1 |
| *Parus cristatus* | 1 |  | 1 | 1 | 1 | 1 |
| *Troglodytes troglodytes* | 1 | 1 |  | 1 | 1 | 1 |
| *Turdus merula* | 1 |  | 1 | 1 | 1 | 1 |
| *Buteo buteo* |  | 1 |  | 1 | 1 | 1 |
| *C. coccothraustes** |  | 1 |  | 1 | 1 | 1 |
| *Columba palumbus* |  | 1 | 1 | 1 | 1 | 1 |
| *Picus canus* |  | 1 |  |  |  |  |
| *Sitta europaea* |  | 1 |  |  |  |  |
| *Cuculus canorus* |  |  | 1 |  |  |  |
| *Garrulus glandarius* |  |  | 1 |  |  |  |
| *Parus montanus* |  |  | 1 |  |  |  |
| Number of species | 7 | 6 | 9 | 9 | 9 | 9 |
| Average sensitivity score | 31.14 | 24.17 | 45.67 | 61.89 | 60.33 | 52.56 |

**Coccothraustes coccothraustes*
