# Supplementary material for: A Niche-Based Framework to Assess Current Monitoring of European Forest Birds and Guide Indicator Species' Selection
Source: PLoS One. 2014 May 12;9(5):e97217. doi: 10.1371/journal.pone.0097217 (PMC4018337; doi:10.1371/journal.pone.0097217)
Supplement: Table S7 — BREAKPOINT sets for the forest-type and regional indicators drawn solely from species currently covered by PECBMS. (DOCX) [file pone.0097217.s014.docx]

**Table S7**: Species included in the *BREAKPOINT* sets drawn only from species currently covered by PECBMS for forest-type specific and regional indicators. Species’ sensitivity scores are calculated as their niche breadth*reliance, with higher values indicating species less sensitive to changes in resource abundance or availability. ‘0/1’ identifies species that were interchangeable in any given breakpoint set due to equal sensitivity scores – see note for further details.

| Species | Conifer-dominated | Broadleaf-dominated | North | South | East | West |
| --- | --- | --- | --- | --- | --- | --- |
| *Accipiter nisus* | 1 |  | 1 | 1 | 1 | 1 |
| *Aegithalos caudatus* | 1 |  |  |  |  |  |
| *Bonasa bonasia* | 1 |  | 1 |  | 1 |  |
| *Dendrocopos major* | 1 |  | 1 | 1 | 1 | 1 |
| *Emberiza rustica* | 1 | 1 | 1 |  |  |  |
| *Nucifraga caryocatactes* | 1 |  | 1 | 1 | 1 | 1 |
| *Parus cristatus* | 1 |  | 1 | 1 | 1 | 1 |
| *Phylloscopus bonelli* | 1 |  |  | 1 |  |  |
| *Phylloscopus sibilatrix* | 1 | 1 | 1 | 1 | 1 | 1 |
| *Regulus ignicapilla* | 1 |  |  |  |  |  |
| *Troglodytes troglodytes* | 1 |  |  | 1 | 1 | 1 |
| *Turdus merula* | 1 |  | 1 | 1 | 1 | 1 |
| *Buteo buteo* |  | 1 |  | 1 | 1 | 1 |
| *C. coccothraustes** |  | 1 |  | 1 | 1 | 0/1^a^ |
| *Dendrocopos medius* |  | 1 |  | 1 | 1 | 1 |
| *Garrulus glandarius* |  | 1 | 1 |  |  | 0/1^a^ |
| *Oriolus oriolus* |  | 1 |  | 1 | 1 | 1 |
| *Parus palustris* |  | 1 |  |  |  |  |
| *Phoenicurus phoenicurus* |  | 1 |  |  |  |  |
| *Picus canus* |  | 1 |  |  |  |  |
| *Pyrrhula pyrrhula* |  | 1 |  | 1 | 1 |  |
| *Columba palumbus* |  |  | 1 |  |  | 1 |
| *Cuculus canorus* |  |  | 1 |  |  |  |
| *Hippolais icterina* |  |  | 1 |  |  |  |
| *Muscicapa striata* |  |  | 1 | 1 | 1 | 1 |
| *Parus ater* |  |  | 1 |  |  | 0/1^a^ |
| *Parus montanus* |  |  | 1 |  |  | 0/1^a^ |
| *Sylvia borin* |  |  | 1 |  |  |  |
| *Dendrocopos minor* |  |  |  |  |  | 0/1^a^ |
| *Columba oenas* |  |  |  | 1 | 1 |  |
| *Ficedula hypoleuca* |  |  |  | 1 |  | 1 |
| *Luscinia megarhynchos* |  |  |  | 1 |  |  |
| *Phylloscopus collybita* |  |  |  | 1 |  |  |
| *Jynx torquilla* |  |  |  |  |  | 1 |
| Number of species | 12 | 11 | 16 | 18 | 15 | 17 |
| Average sensitivity score | 20.50 | 11.45 | 29.50 | 34.28 | 35.67 | 31.12 |

**Coccothraustes coccothraustes*

^a^Either *Parus ater*, *Garrulus glandarius & Parus montanus;* *Coccothraustes coccothraustes, Dendrocopos minor &* *Parus ater;* or *Dendrocopus minor, Parus montanus* & *Garrulus glandarius* could be included
