# Supplementary material for: A Niche-Based Framework to Assess Current Monitoring of European Forest Birds and Guide Indicator Species' Selection
Source: PLoS One. 2014 May 12;9(5):e97217. doi: 10.1371/journal.pone.0097217 (PMC4018337; doi:10.1371/journal.pone.0097217)
Supplement: Table S8 — SENSITIVE sets for the pan-European, forest-type and regional indicators drawn solely from species currently covered by PECBMS. (DOCX) [file pone.0097217.s015.docx]

**Table S8**: Species included in the *SENSITIVE* sets drawn only from species currently covered by PECBMS for the main pan-European, forest-type specific and regional indicators. Species’ sensitivity scores are calculated as their niche breadth*reliance, with higher values indicating species less sensitive to changes in resource abundance or availability.

| Species | Main | Conifer-dominated | Broadleaf-dominated | North | South | East | West |
| --- | --- | --- | --- | --- | --- | --- | --- |
| *Accipiter nisus* | 1 | 1 | 1 | 1 | 1 | 1 | 1 |
| *Anthus trivialis* | 1 | 1 |  |  | 1 | 1 | 1 |
| *Bonasa bonasia* | 1 | 1 |  | 1 |  | 1 |  |
| *Buteo buteo* | 1 |  | 1 |  | 1 | 1 | 1 |
| *Carduelis spinus* | 1 | 1 | 1 | 1 |  | 1 | 1 |
| *C. coccothraustes** | 1 |  | 1 |  | 1 | 1 | 1 |
| *Columba oenas* | 1 |  |  |  | 1 | 1 | 1 |
| *Dendrocopos major* | 1 | 1 |  | 1 | 1 | 1 | 1 |
| *Dendrocopos medius* | 1 |  | 1 |  | 1 | 1 | 1 |
| *Dendrocopos minor* | 1 |  | 1 |  | 1 | 1 | 1 |
| *Emberiza rustica* | 1 | 1 | 1 | 1 |  |  |  |
| *Ficedula albicollis* | 1 |  | 1 |  |  | 1 |  |
| *Ficedula hypoleuca* | 1 |  | 1 |  | 1 | 1 | 1 |
| *Hippolais icterina* | 1 |  | 1 | 1 |  |  | 1 |
| *Jynx torquilla* | 1 |  | 1 |  | 1 | 1 | 1 |
| *Luscinia megarhynchos* | 1 |  | 1 |  | 1 | 1 |  |
| *Muscicapa striata* | 1 |  | 1 | 1 | 1 | 1 | 1 |
| *Nucifraga caryocatactes* | 1 | 1 |  | 1 | 1 | 1 | 1 |
| *Oriolus oriolus* | 1 |  | 1 |  | 1 | 1 | 1 |
| *Parus ater* | 1 | 1 |  | 1 | 1 | 1 | 1 |
| *Parus cristatus* | 1 | 1 |  | 1 | 1 | 1 | 1 |
| *Phylloscopus bonelli* | 1 | 1 | 1 |  | 1 |  |  |
| *Phylloscopus sibilatrix* | 1 | 1 | 1 | 1 | 1 | 1 | 1 |
| *Pyrrhula pyrrhula* | 1 | 1 | 1 | 1 | 1 | 1 | 1 |
| *Regulus ignicapilla* | 1 | 1 |  |  | 1 | 1 | 1 |
| *Regulus regulus* | 1 | 1 | 1 | 1 | 1 | 1 | 1 |
| *Sitta europaea* | 1 |  | 1 |  | 1 |  | 1 |
| *Sylvia borin* | 1 |  | 1 | 1 | 1 | 1 | 1 |
| *Troglodytes troglodytes* | 1 | 1 |  |  | 1 | 1 | 1 |
| *Turdus merula* | 1 | 1 |  | 1 | 1 | 1 | 1 |
| *Aegithalos caudatus* |  | 1 |  |  | 1 |  |  |
| *Certhia familiaris* |  | 1 | 1 | 1 | 1 | 1 | 1 |
| *Dryocopus martius* |  | 1 |  | 1 | 1 | 1 | 1 |
| *Lullula arborea* |  | 1 |  |  | 1 | 1 |  |
| *Phylloscopus trochilus* |  | 1 |  |  | 1 | 1 | 1 |
| *Serinus serinus* |  | 1 |  |  |  |  |  |
| *Garrulus glandarius* |  |  | 1 | 1 | 1 |  | 1 |
| *Parus palustris* |  |  | 1 |  |  |  |  |
| *Phoenicurus phoenicurus* |  |  | 1 |  |  |  |  |
| *Picus canus* |  |  | 1 | 1 | 1 | 1 |  |
| *Carduelis flammea* |  |  |  | 1 |  |  |  |
| *Cuculus canorus* |  |  |  | 1 |  |  |  |
| *Parus montanus* |  |  |  | 1 |  |  |  |
| *Turdus philomelos* |  |  |  | 1 |  |  |  |
| *Turdus viscivorus* |  |  |  | 1 |  |  |  |
| *Certhia brachydactyla* |  |  |  |  | 1 |  | 1 |
| *Parus caeruleus* |  |  |  |  | 1 |  |  |
| *Phylloscopus collybita* |  |  |  |  | 1 |  | 1 |
| *Picus viridis* |  |  |  |  | 1 |  | 1 |
| Number of species | 30 | 22 | 25 | 24 | 36 | 31 | 32 |
| Average sensitivity score | 19.47 | 17.68 | 8.36 | 26.17 | 25.75 | 26.03 | 25.16 |

**Coccothraustes coccothraustes*
